# Multiple Roles of Actin in Exo- and Endocytosis

**DOI:** 10.3389/fnsyn.2022.841704

**Published:** 2022-03-04

**Authors:** Ling-Gang Wu, Chung Yu Chan

**Affiliations:** National Institute of Neurological Disorders and Stroke, Bethesda, MD, United States

**Keywords:** actin, exocytosis, endocytosis, synaptic transmission, neurological disorder

## Abstract

Cytoskeletal filamentous actin (F-actin) has long been considered a molecule that may regulate exo- and endocytosis. However, its exact roles remained elusive. Recent studies shed new light on many crucial roles of F-actin in regulating exo- and endocytosis. Here, this progress is reviewed from studies of secretory cells, particularly neurons and endocrine cells. These studies reveal that F-actin is involved in mediating all kinetically distinguishable forms of endocytosis, including ultrafast, fast, slow, bulk, and overshoot endocytosis, likely *via* membrane pit formation. F-actin promotes vesicle replenishment to the readily releasable pool most likely *via* active zone clearance, which may sustain synaptic transmission and overcome short-term depression of synaptic transmission during repetitive firing. By enhancing plasma membrane tension, F-actin promotes fusion pore expansion, vesicular content release, and a fusion mode called shrink fusion involving fusing vesicle shrinking. Not only F-actin, but also the F-actin assembly pathway, including ATP hydrolysis, N-WASH, and formin, are involved in mediating these roles of exo- and endocytosis. Neurological disorders, including spinocerebellar ataxia 13 caused by Kv3.3 channel mutation, may involve impairment of F-actin and its assembly pathway, leading in turn to impairment of exo- and endocytosis at synapses that may contribute to neurological disorders.

## Introduction

Vesicle exocytosis releases neurotransmitters and hormones to mediate important functions, such as synaptic transmission, stress responses, and immune responses (Wu et al., 2014; Chang et al., 2017; Brunger et al., 2018; Sharma and Lindau, 2018). After exocytosis, fused vesicles must be retrieved *via* endocytosis, which recycles vesicles and thus sustains exocytosis in secretory cells, particularly in nerve terminals (Wu et al., 2014; Kononenko and Haucke, 2015; Gan and Watanabe, 2018). Half a century of studies identified many core exo- and endocytic proteins, such as SNARE proteins, synaptotagmin, and dynamin (Jahn and Fasshauer, 2012; Kaksonen and Roux, 2018; Mettlen et al., 2018). However, the role of actin in the exo- and endocytosis of secretory cells remained not well understood despite it being one of the most abundant cytoskeletal proteins (Cingolani and Goda, 2008; Li et al., 2018).

Studies over the past three decades led to proposals that actin is involved in many steps of exo- and endocytosis in secretory cells. These potential roles include vesicle clustering in nerve terminals, physical barrier to prevent vesicle docking at the plasma membrane, facilitation of vesicle mobilization to the readily releasable pool (RRP) that seems to contradict its physical barrier function, fusion pore expansion, vesicle merging at the plasma membrane, and endocytosis that recycles vesicles (Cingolani and Goda, 2008; Li et al., 2018). With respect to its vesicle clustering role, filamentous actin (F-actin), together with synapsin, has long been proposed to provide a cytoskeletal scaffold linking vesicles together, leading to formation of vesicle clusters in nerve terminals (reviewed in Cingolani and Goda, 2008). Recent studies suggest that synapsin alone can form a distinct liquid phase in an aqueous environment, which may catalyze vesicle clustering at nerve terminals (Milovanovic et al., 2018). Microinjection of reagents that bind to the intrinsically disordered region of synapsin causes dispersal of synaptic vesicle clusters, suggesting that liquid-liquid phase separation may mediate synaptic vesicle clustering (Pechstein et al., 2020). These results suggest re-examination of the role of F-actin in vesicle clustering in the future. Accordingly, this topic will not be further discussed here.

With respect to its physical barrier role, early studies showed that disruption of actin polymerization increases the frequency of spontaneous and evoked transmitter release at synapses and in endocrine cells, suggesting that F-actin behind the plasma membrane may restrain docking of vesicles at release sites (Aunis and Bader, 1988; Morales et al., 2000; Trifaro et al., 2000; Chowdhury et al., 2002; Malacombe et al., 2006; Cingolani and Goda, 2008). In neuroendocrine cells, the region of cytosol adjacent to the plasma membrane contains the most actin filaments, called the cortical actin network (reviewed in Meunier and Gutierrez, 2016). This cortical actin network may act as a physical barrier that opposes vesicle access to release sites, as shown in studies of chemicals that stabilize or inhibit actin polymerization (Wen et al., 2011; Meunier and Gutierrez, 2016). The physical barrier function has been systematically surveyed in several excellent reviews (Meunier and Gutierrez, 2016; Papadopulos, 2017; Li et al., 2018). Thus, readers are referred to these reviews for more detailed discussion of the physical barrier function. This review focuses on actin’s roles in mediating endocytosis, facilitating replenishment of the RRP, promoting fusion pore expansion, and merging vesicles with the plasma membrane. Potential mechanisms that may reconcile the apparent conflict between actin’s physical barrier function versus facilitatory role in RRP replenishment are also suggested.

## Actin Is Crucial for All Kinetically Distinguishable Forms of Endocytosis

Early studies regarding actin’s role in endocytosis in secretory cells reached different conclusions as to whether actin is needed and at which step(s). For example, at lamprey giant synapses, latrunculin B, which disrupts actin polymerization, causes accumulation of clathrin-coated pits and vesicles (Shupliakov et al., 2002; Bourne et al., 2006), whereas latrunculin A (Lat A), which also disrupts actin polymerization, does not affect endocytosis as measured using FM1-43 uptake (Bleckert et al., 2012). At frog neuromuscular junctions, Lat A reduces FM1-43 uptake into nerve terminals (Richards et al., 2004). Whether this effect is caused by inhibition of endocytosis or exocytosis has not been distinguished. On the other hand, cytochalasin D, which inhibits actin polymerization, does not affect FM1-43 uptake or release, implying that actin may not be needed for endocytosis at frog neuromuscular junctions (Betz and Henkel, 1994). At hippocampal synapses, Lat A does not affect endocytosis after action potential trains (Li and Murthy, 2001; Sankaranarayanan et al., 2003; Hua et al., 2011). At goldfish retinal bipolar synapses, latrunculin B and cytochalasin D do not inhibit endocytosis over a time course <∼20 s, but may slow down bulk endocytosis (Holt et al., 2003). Lat A inhibits fast endocytosis at mossy fiber boutons (Delvendahl et al., 2016) and ultrafast endocytosis at hippocampal synapses (Watanabe et al., 2014). These pharmacological studies do not reach a consensus regarding the effect of actin blockers on endocytosis at the same synapse, at different synapses, or for different endocytic modes. However, these studies rely on actin blockers, and some studies use only one blocker at one concentration. False-positive or -negative results owing to off-target effects or difficulty blocking actin polymerization in live cells might contribute to these apparent controversial results (Bleckert et al., 2012). In the following, we review more recent studies that used genetic combined with pharmacological approaches to study actin’s endocytic role in mammalian cells.

**FIGURE 1:**
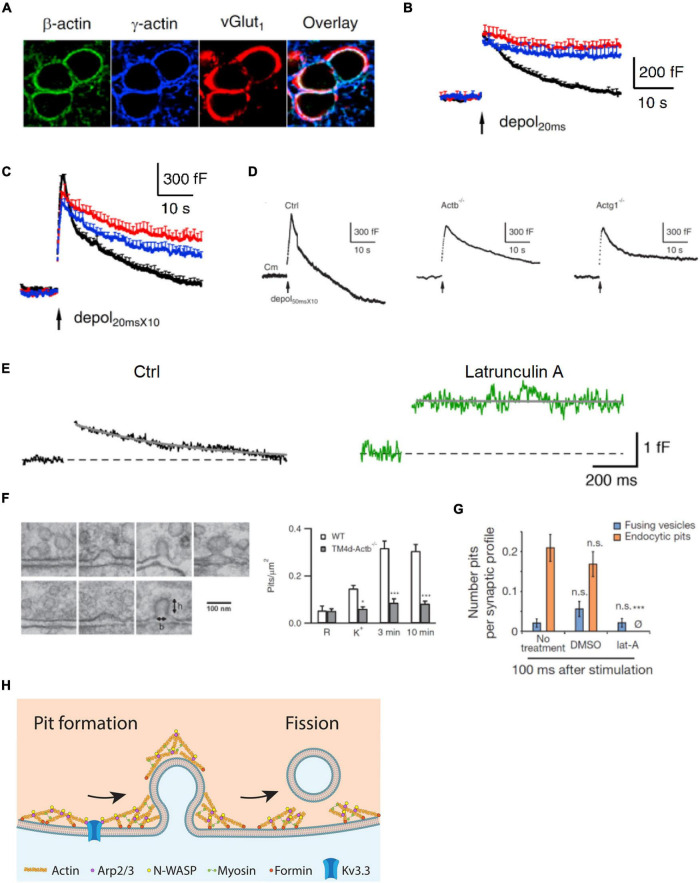
Actin is involved in mediating ultrafast, fast, slow, bulk, and overshoot endocytosis at synapses likely by facilitating membrane pit formation. **(A)** Antibody staining of β-actin, γ-actin, and vesicular glutamate transporter 1 (vGluT_1_) in calyx of Held nerve terminals. **(B)** Actin involvement in slow endocytosis: mean capacitance (Cm) traces (mean + SEM) induced by a 20 ms depolarization from –80 to +10 mV (depol_20 ms_, arrow) in calyces of wild-type (black), Actb^–/–^ (red), and Actg1^–/–^ (blue) mice. Depol_20 ms_ induces slow endocytosis in wild-type calyces. **(C)** Actin involvement in rapid (or fast) endocytosis: similar arrangement as in **B** except that the stimulus was 10 depol_20 ms_ at 10 Hz (depol_20 msX10_), which induces rapid (or fast) endocytosis in wild-type calyces. **(D)** Actin involvement in bulk endocytosis and endocytosis overshoot: sampled Cm induced by depol_50 msX10_ (10 depol_50 ms_ at 10 Hz) with 5.5 mM calcium in the bath from wild-type (Ctrl), β-actin (Actb)^–/–^, and γ-actin (Actg1)^–/–^ calyces. Depol_50 msX10_ induces bulk endocytosis (a large step of downward capacitance shift) and endocytosis overshoot in Ctrl. **(E)** Actin involvement in very fast endocytosis: averaged Cm response to single action potentials in Ctrl (black) and in the presence of latrunculin A (Lat A, green). Gray solid lines are exponential fits to the Cm decay. **(F)** Left: electron microscopy images of membrane pits of various shapes obtained during or after high potassium chloride (KCl) application from either wild-type (WT) or Actb^–/–^ hippocampal cultures. Right: the number of pits before (R) and after KCl application (K^+^, 0 min; 3 min and 10 min) in wild-type (WT) control and Actb^–/–^ hippocampal synapses (mean + SEM). *p < 0.05; ***p < 0.001 (t-test). The data show that β-actin knockout inhibits pit formation. **(G)** Actin involvement in ultrafast pit formation: average number of exocytic pits (blue) and endocytic pits (orange) in cells treated with latrunculin A (Lat A) or dimethyl sulfoxide (DMSO). ***p < 0.001 (t-test). **(H)** Schematic diagram showing the involvement of F-actin and its nucleation factors, such as Kv3.3 potassium channel, Arp2/3, formin, and myosin II in all kinetically distinguishable forms of endocytosis, including ultrafast, fast, slow, bulk, and overshoot endocytosis. Panels **A–D,F** are adapted from Wu et al. (2016) with permission. Panel **E** is adapted from Delvendahl et al. (2016) with permission. Panel **G** is adapted from Watanabe et al. (2013) with permission.

There are six actin isoforms in mammalian cells. β-Cytoplasmic actin (β-actin), encoded by *Actb*, and γ-cytoplasmic actin (γ-actin), encoded by *Actg1*, are ubiquitously expressed and are the major isoforms in the nervous system (Figure 1A; Herman, 1993; Cheever and Ervasti, 2013). β- or γ-actin knockout at two types of nerve terminals in mice, the calyx of Held and hippocampal boutons, were generated (Wu et al., 2016). At the giant calyx of Held nerve terminal, capacitance measurements reveal four kinetically different forms of endocytosis, including slow endocytosis (tens of seconds), rapid or fast endocytosis (a few seconds), bulk endocytosis (forming vesicles larger than regular vesicles), and endocytosis overshoot (retrieving more vesicles than were exocytosed) (Sun and Wu, 2001; Sun et al., 2002; Yamashita et al., 2005, 2010; Renden and Von Gersdorff, 2007; Xu et al., 2008; Hosoi et al., 2009; Wu et al., 2009; Xue et al., 2012). At cultured small conventional hippocampal boutons, endocytosis can be measured with imaging of pH-sensitive pHluorin attached to synaptic vesicle proteins (Sankaranarayanan and Ryan, 2000; Wienisch and Klingauf, 2006; Balaji and Ryan, 2007; Sun et al., 2010; Zhang et al., 2013; Kavalali and Jorgensen, 2014; Chanaday and Kavalali, 2018).

Capacitance measurements at calyces reveal that β- or γ-actin knockout inhibits slow endocytosis, rapid endocytosis (Figures 1B,C), bulk endocytosis (detected as the large downward capacitance shift; Figure 1D), and endocytosis overshoot (Figure 1D; Wu et al., 2016). Electron microscopy and pHluorin imaging at hippocampal synapses show that β- or γ-actin knockout inhibits slow endocytosis and endosome-like structure formation due to bulk endocytosis (Wu et al., 2016). Thus, actin is crucial in mediating rapid, slow, bulk, and overshoot endocytosis. An actin mutant with a polymerization defect could not rescue endocytosis in boutons lacking β-actin, suggesting that polymerized actin, which is known to exert mechanical forces (Mund et al., 2018), is involved in generating forces needed for endocytosis (Wu et al., 2016). Actin knockout does not affect the rate of fission pore closure during bulk endocytosis at calyces but inhibits membrane pit formation at hippocampal synapses (Figure 1F), suggesting that F-actin may exert mechanical force to bend membrane and thereby generate membrane pits (Wu et al., 2016). Taken together, these findings indicate that polymerized actin may mediate rapid, slow, bulk, and overshoot endocytosis by providing mechanical forces to bend membrane (Wu et al., 2016).

This suggestion, supported by genetic evidence, is consistent with pharmacological evidence that Lat A inhibits a very fast form of endocytosis with a time constant of ∼0.5 s recorded with capacitance measurements at cerebellar and hippocampal mossy fiber boutons (Figure 1E; Delvendahl et al., 2016). Lat A also inhibits ultrafast pit formation detected with electron microscopy combined with rapid freezing after optogenetic stimulation at hippocampal synapses (Figure 1G), which suggests involvement of F-actin in ultrafast endocytosis (Watanabe et al., 2013). Taken together, these results indicate that F-actin may be involved in mediating all distinguishable forms of endocytosis at synapses, including ultrafast, fast, slow, bulk, and overshoot endocytosis *via* its role in pit generation (Figure 1H).

This possibility, inferred from studies of secretory cells, may not generalize to non-secretory vesicle endocytosis in mammalian cells, where F-actin is thought to be needed to overcome membrane tension only when plasma membrane tension is high (Merrifield et al., 2005; Yarar et al., 2005; Ferguson et al., 2009; Saffarian et al., 2009; Boulant et al., 2011). However, it is consistent with actin’s role during endocytosis in yeast, which possess a cell wall, where actin polymerization from the plasma membrane toward the cytosol has been suggested to generate a force pushing and elongating the neck, forming a tube-shape pit (Picco et al., 2015; Kaksonen and Roux, 2018; Mund et al., 2018). Whether this mechanism applies to mammalian cells, which do not contain cell walls, remains to be determined. Imaging F-actin dynamics during endocytic membrane pit formation in real time, a very recent study in mammalian chromaffin cells suggests that F-actin may providing a point-pulling force at the center of the endocytic zone to pull membrane inward, forming membrane pits together with dynamin (Shin et al., 2022).

Many factors are involved in nucleating F-actin. Neural Wiskott-Aldrich-syndrome protein (N-WASP) may activate actin-related protein 2/3 complex (Arp2/3) to form branched actin networks together with myosin, whereas formins are involved in forming linear actin filaments (Cingolani and Goda, 2008; Kononenko and Haucke, 2015; Kaksonen and Roux, 2018). Myosin II inhibitor blebbistatin suppresses endocytosis at hippocampal and calyx of Held synapses, suggesting that myosin II-dependent F-actin nucleation is involved in mediating endocytosis (Yue and Xu, 2014; Soykan et al., 2017). Consistent with this suggestion, knockout of myosin IIB or application of blebbistatin reduces horseradish peroxidase uptake that may reflect endocytosis at hippocampal synapses (Chandrasekar et al., 2013). To investigate the function of actomyosin, actin nucleation, which precedes F-actin assembly, has been studied with pharmacological approaches and gene knockdown (Soykan et al., 2017). Inhibition of formin-mediated assembly of linear actin filaments by the selective inhibitor SMIFH2 (Ganguly et al., 2015) in turn inhibits endocytosis at hippocampal and calyx-type synapses (Soykan et al., 2017). Similarly, shRNA-mediated knockdown of the diaphanous-related formin mDia1 slows synaptic vesicle endocytosis (Soykan et al., 2017). These results suggest that formin-dependent actin filament assembly may regulate synaptic vesicle endocytosis (Soykan et al., 2017).

Recent studies reveal an unexpected relationship between potassium channels and endocytosis mediated by nucleation of F-actin at synapses (Zhang et al., 2016; Wu et al., 2021). Since the discovery of potassium channels, the physiological and pathological impact of potassium channels has been attributed to their ion conductance, which sets the cell membrane potential and repolarizes the membrane during action potentials (Kaczmarek and Zhang, 2017). For example, Kv3 family channels are generally considered to regulate neurotransmitter release by repolarizing the membrane during action potentials (Kaczmarek and Zhang, 2017). A recent study reports a crucial function of these channels independent of their ion conductance – by organizing the F-actin cytoskeleton in nerve terminals, Kv3.3 protein facilitates rapid and slow endocytosis at hippocampal and calyx of Held synapses (Wu et al., 2021). The extended cytoplasmic C-terminal domain of Kv3.3 at the plasma membrane binds to and thereby recruits beneath the plasma membrane the Arp2/3, which is involved in nucleating the cortical F-actin cytoskeleton (Zhang et al., 2016; Wu et al., 2021). The channel mutation G592R Kv3.3 causes Kv3.3 to fail to bind Arp2/3, and thus disrupts the ability of the channel to nucleate F-actin in nerve terminals, resulting in inhibition of synaptic vesicle endocytosis (Zhang et al., 2016; Wu et al., 2021). Since this mutation may cause spinocerebellar ataxia 13 (Middlebrooks et al., 2013), inhibition of synaptic vesicle endocytosis by disruption of F-actin nucleation may contribute to the generation of spinocerebellar ataxia 13 (Wu et al., 2021).

Figure 1H provides a schematic summary of the role of actin and its assembly pathways in mediating synaptic vesicle endocytosis. The implications of the studies discussed above are integrated in the figure to show the involvement of F-actin and F-actin nucleation factors, such as Kv3.3, Arp2/3, formin, and myosin II, in all kinetically distinguishable forms of endocytosis.

## Actin Facilitates Vesicle Replenishment to the Readily Releasable Pool

**FIGURE 2:**
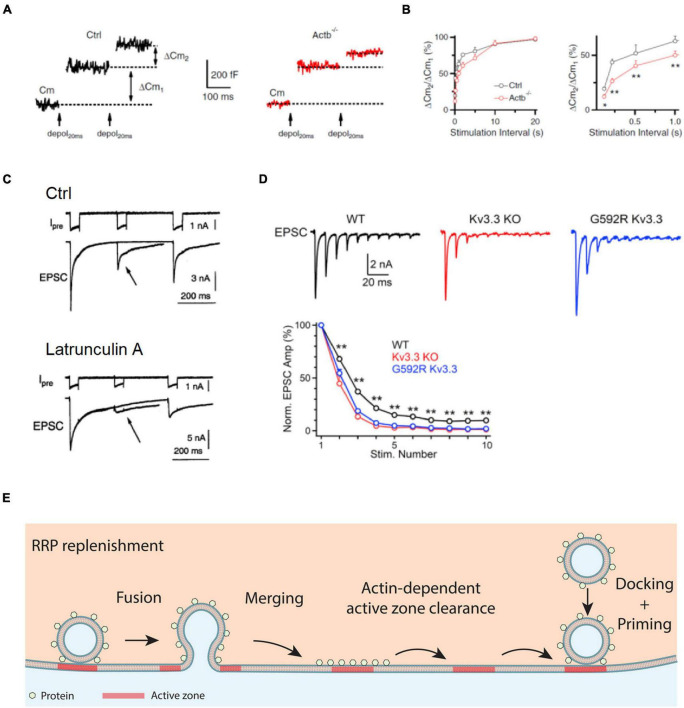
Actin promotes RRP replenishment likely by facilitating active zone clearance. **(A)** Sampled Cm traces induced by a pair of depol_20 ms_ at an interval of 200 ms in a control (Ctrl, left) and an Actb^–/–^ (right) calyx. Measurements of the capacitance jumps induced by the first and second depol_20 ms_ (ΔCm_1_ and ΔCm_2_) are schematically shown. **(B)** Left: the ratio between the second and first ΔCm (ΔCm_2_/ΔCm_1_) during a pair of depol_20 ms_ plotted versus paired-pulse interval at control and Actb^–/–^ calyces. Right: same as in left but plotting the interval between 0 and 1 s. *p < 0.05; **p < 0.01 (*t*-test). The data in **A,B** show that β-actin knockout reduces ΔCm2/ΔCm1. **(C)** Top: a dual pulse of 50 ms (to 0 mV) was applied at different intervals (200 and 500 ms) to the calyx. Presynaptic calcium currents (Ipre) and excitatory postsynaptic currents (EPSCs) are shown. Bottom: similar arrangement as in the top, but with the presynaptic pipette solution containing Lat A to inhibit actin polymerization. Arrows indicate that latrunculin A inhibits EPSC induced by a second pulse. **(D)** The Kv3.3 channel regulates EPSCs during repetitive action potential firing: sampled EPSCs (top) and the amplitude of EPSCs (bottom, mean + SEM) induced by 10 action potentials at 100 Hz at the calyx of wild-type mice, Kv3.3^–/–^ mice, and mice with a mutation (Kv3.3 G592R) that causes spinocerebellar ataxia 13 and inhibits F-actin nucleation at the calyx. **(E)** Schematic diagram showing that the F-actin cytoskeleton may facilitate active zone clearance and thus RRP replenishment. RRP replenishment may involve active zone clearance, vesicle docking, and vesicle priming that makes the docked vesicle release-ready. Panels **A,B** are adapted from Wu et al. (2016) with permission. Panel **C** is adapted from Sakaba and Neher (2003) with permission. Panel **D** is adapted from Wu et al. (2021) with permission.

After release of vesicles at active zone release sites, they must be replenished. This process, called vesicle mobilization or replenishment to the RRP, is crucial to sustain synaptic transmission and minimize short-term synaptic depression during repetitive firing (Wang and Kaczmarek, 1998; Schneggenburger et al., 1999; Wu and Borst, 1999; Sakaba and Neher, 2001; Von Gersdorff and Borst, 2002; Zucker and Regehr, 2002; Xu and Wu, 2005; He et al., 2009). At the calyx of Held synapse, the rate of RRP replenishment can be measured experimentally with a pair of depolarizing pulses of ∼20–50 ms, each of which can completely deplete the RRP (Figures 2A–C; Wu and Borst, 1999; Sakaba and Neher, 2001; Wu et al., 2016). The exocytosis induced by the second pulse applied at various intervals after the first pulse may thus indicate the time course of RRP replenishment (Figures 2A–C). It has been found that the RRP replenishment is suppressed by a variety of manipulations that inhibit F-actin, including knockout of the β-actin or γ-actin gene (Figures 2A,B), Lat A that inhibits F-actin polymerization (Figure 2C), Kv3.3 that disrupts F-actin nucleation, and mutation of Kv3.3 that causes spinocerebellar ataxia and inhibits F-actin nucleation at nerve terminals (Sakaba and Neher, 2003; Wu et al., 2016, 2021). These results suggest that F-actin facilitates RRP replenishment and impact F-actin nucleation may contribute to the generation of neurological disorders.

Since RRP replenishment sustains synaptic transmission during repetitive firing, suppression of RRP replenishment by inhibition of F-actin predicts severe short-term depression of synaptic transmission. This prediction was verified by measurements of the excitatory postsynaptic current (EPSC) during repetitive firing in Kv3.3 knockout or G592R Kv3.3 knock-in mice, in which F-actin nucleation at nerve terminals is impaired (Figure 2D; Wu et al., 2021). Taken together, these results suggest that F-actin opposes short-term depression, and thus helps to sustain synaptic transmission during repetitive firing.

Given that inhibition of endocytosis by the block of dynamin, calmodulin, calcium influx, and SNARE proteins slows down RRP replenishment, it has been suggested that endocytosis facilitates RRP replenishment *via* clearance of the active zone as perturbed by exocytosis (Hosoi et al., 2009; Wu et al., 2009, 2014; Neher, 2010; Sun et al., 2010; Hua et al., 2013; Xu et al., 2013). Accordingly, actin involvement in endocytosis and RRP replenishment suggests that F-actin promotes RRP replenishment by facilitating active zone clearance (Figure 2E; Wu et al., 2016). Therefore, impairment of RRP replenishment may contribute to the generation of spinocerebellar ataxia 13 and other neurological disorders caused by impairments of F-actin nucleation or assembly.

The exact mechanism of how actin and endocytosis mediate active zone clearance remains unclear. Annexin A2, a calcium-, actin-, and lipid-binding protein involved in exocytosis, may induce actin bundling that seems essential for generating active exocytotic sites in chromaffin cells (Gabel et al., 2015). Such a mechanism might provide hints about how F-actin facilitates replenishment of the RRP.

Actin’s role in facilitating RRP replenishment is apparently in conflict with its physical barrier function, which has been suggested based on an observed increase in spontaneous and evoked vesicular content release after inhibition of actin polymerization (Aunis and Bader, 1988; Morales et al., 2000; Trifaro et al., 2000; Chowdhury et al., 2002; Malacombe et al., 2006; Cingolani and Goda, 2008). This discrepancy could be reconciled if the RRP replenishment reflects mostly the active zone clearance, but not solely just vesicle movement to the docking site. The RRP is functionally defined as a pool of vesicles that can be depleted by a brief depolarization (Von Gersdorff and Borst, 2002; Neher and Sakaba, 2008; Wu et al., 2014). For example, at the calyx of Held, the RRP is defined as the vesicles being released by a 20 or 50 ms depolarization or about 20 action potentials at 100–300 Hz (Wang and Kaczmarek, 1998; Schneggenburger et al., 1999; Wu and Borst, 1999; Sakaba and Neher, 2001; Sun and Wu, 2001). The number of vesicles in the RRP, estimated with capacitance measurements (Sun and Wu, 2001; Sun et al., 2002), could be larger than the number of morphologically docked vesicles observed with electron microscopy (Sätzler et al., 2002; Taschenberger et al., 2002). Thus, RRP replenishment may reflect not only physical movement of vesicles from the cytosol to the plasma membrane docking site, but also the summed activity of active zone clearance, vesicle docking, and subsequent vesicle priming to become release-ready. Accordingly, we suggest that within the time scale of seconds after RRP depletion, F-actin cytoskeleton may help in active zone clearance that facilitates RRP replenishment. In a longer time scale and likely a longer distance, F-actin cytoskeleton may serve as a physical barrier for vesicles deep inside the cytosol to move toward the plasma membrane. This suggestion may reconcile the apparent controversy surrounding F-actin regarding its facilitatory role in RRP replenishment and its inhibitory function in vesicle movement toward the plasma membrane. Verifying this suggestion in the future may require imaging and quantification of individual vesicle movements toward, and fusion at, the plasma membrane release site in live cells.

## Filamentous Actin Promotes Fusion Pore Expansion and Thus Content Release by Enhancing Membrane Tension

**FIGURE 3:**
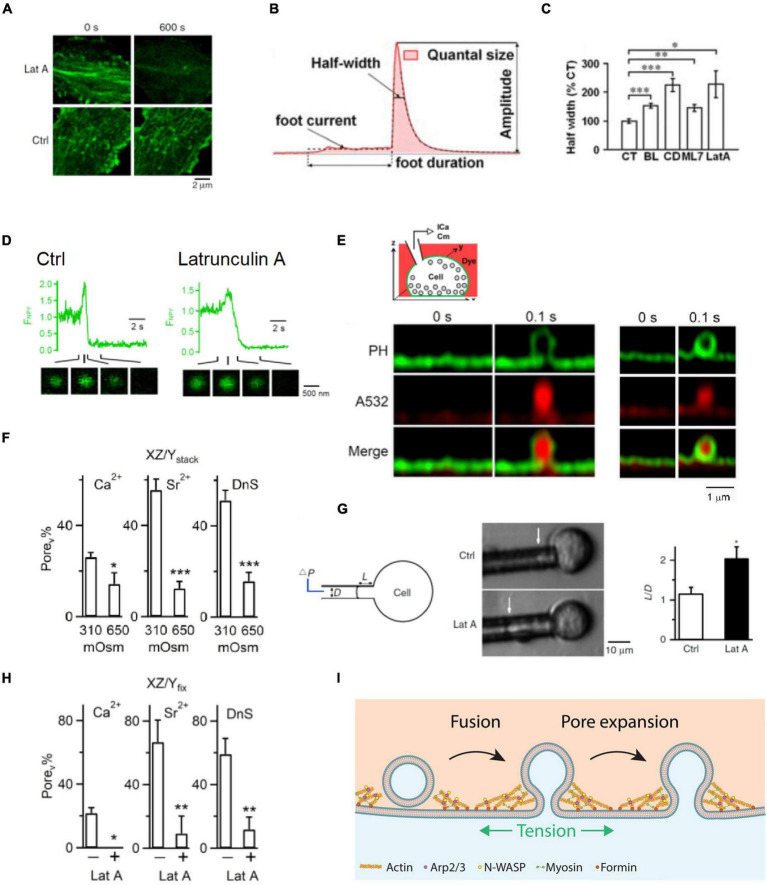
F-actin promotes fusion pore expansion by enhancing plasma membrane tension. **(A)** Latrunculin A (Lat A) reduces F-actin: sampled Lifeact-labeled F-actin at the cell bottom of bovine adrenal chromaffin cells before (0 s, left) and 600 s after (right) application of Lat A (3 μM) or a control (Ctrl) solution. **(B)** A single amperometric spike along with the five parameters: quantal size Q (pC), half-width (ms), peak amplitude (pA), foot signal duration (ms), and mean foot current (pA). **(C)** Half-width (mean ± SEM) of amperometric spikes in control (CT) or in the presence of blebbistatin (BL), cytochalasin D (CD), ML-7 (ML7), or Lat A. Data normalized to the mean in control. *p < 0.05; ^**^p < 0.01; ^***^p < 0.001 (*t*-test). **(D)** Lat A slows down neuropeptide Y-EGFP (NPY-EGFP) release from single vesicles: fluorescence of NPY-EGFP (F_NPY_) from single vesicles in Ctrl (left) and in the presence of Lat A (right). Decay indicates release of neuropeptide Y-EGFP. NPY-EGFP images at times indicated are also shown. The initial increase of NPY-EGFP fluorescence is due to fusion pore opening that increases the vesicular lumen pH. **(E)** Top: setup drawing. Cell membrane is labeled with the phospholipase C ΔPH domain attached with mNeonGreen (PH_G_, green), whereas the bath solution contains Atto 532 (A532, red, pseudo-color). Bottom left: STED PH_G_/A532 images immediately before (time 0) and after fusion during imaging every 0.1 s. PH_G_-labeled fusion pore is visible. The stimulation was a depolarization from –80 to +10 mV for 1 s (depol_1 s_). Bottom right: similar to left panel but showing a pore not visible to STED microscopy. **(F)** The percentage of fusion pores induced by depol_1s_ that are visible to STED microscopy (Pore_v_%) at 310 or 650 mOsm (bath solution) in calcium (Ca^2+^), strontium (Sr^2+^), or dynasore (DnS, with 5 mM Ca^2+^). Pore_v_ was detected with PH_G_ STED imaging as shown in **E**. Data show that 650 mOsm reduces Pore_v_%. **(G)** Latrunculin A (Lat A) reduces membrane tension. Left: drawings of the micropipette aspiration technique. Negative pressure (ΔP) in the pipette (with a diameter D) draws the cell membrane into the pipette by a length L. Middle: pipette-aspirated cells (bright-field images) in the absence (Ctrl) and presence of Lat A (0.5 μM). Arrows indicate membrane projection (L) into the micropipette (ΔP = 500 Pa). Right: normalized projection length (L/D, mean + SEM) for aspirated cells in the absence (Ctrl) or presence of Lat A (0.5 μM). *p = 0.011 (*t*-test). **(H)** Lat A inhibits Pore_v_ (fusion pore visible to STED microscopy) percentage: the percentage of Pore_v_ (Pore_v_%) in the absence (–) or presence (+) of 3 μM Lat A in a bath containing Ca^2+^, Sr^2+^, or DnS. Pore_v_ was detected with STED imaging of PH_G_ as shown in panel **E**. **(I)** Schematic drawing showing that F-actin cytoskeleton enhances plasma membrane tension and thus promotes fusion pore expansion that releases vesicular contents rapidly and completely. Promotion of fusion pore expansion does not necessarily flatten the fusion pore, explained in more detail in Figure 4. Panels **A,D,G** are adapted from Wen et al. (2016) with permission. Panels **B,C** are adapted from Berberian et al. (2009) with permission. Panel **E,F,H** are adapted from Shin et al. (2018) with permission.

Filamentous actin blockers that reduce F-actin (Figure 3A) and myosin II inhibitors slow down catecholamine release as detected with amperometry (Figures 3B,C; Neco et al., 2008; Berberian et al., 2009; Olivares et al., 2014) and prolong release of vesicular lumen protein neuropeptide Y-EGFP as detected with imaging in chromaffin cells (Figure 3D; Wen et al., 2016). These results suggest that F-actin speeds up vesicular content release in chromaffin cells. How does F-actin facilitate vesicular content release? Recent studies addressed this question by direct visualization of fusion pore opening, expansion, constriction and closure with super-resolution stimulated emission depletion (STED) microscopy at a neuroendocrine cell, the adrenal chromaffin cell containing ∼180–720 nm diameter vesicles (Figure 3E; Wen et al., 2016; Zhao et al., 2016; Shin et al., 2018). It has been observed that fusion pore size may vary between 0 and 490 nm within 26 ms to seconds (Shin et al., 2018). These pore dynamics are crucial in determining the efficiency of vesicular cargo release and vesicle retrieval (Shin et al., 2018). They are generated by competition between mechanisms for pore expansion and mechanisms for pore constriction and closure (Shin et al., 2018). Increasing the extracellular solution osmolarity, which shrinks the cell size and thus may reduce plasma membrane tension (Wen et al., 2016), reduces the initial fusion pore size as measured with STED microscopy (Figure 3F; Shin et al., 2018). Lat A, which reduces F-actin and plasma membrane tension as measured with the pipette aspiration technique in chromaffin cells (Figure 3G; Wen et al., 2016), also reduces the initial fusion pore size as measured with STED microscopy (Figure 3H; Shin et al., 2018). Thus, F-actin promotes fusion pore expansion by enhancing plasma membrane tension (Shin et al., 2018), explaining why F-actin facilitates vesicular content release (Figure 3I).

## Filamentous Actin Promotes Shrink Fusion

**FIGURE 4:**
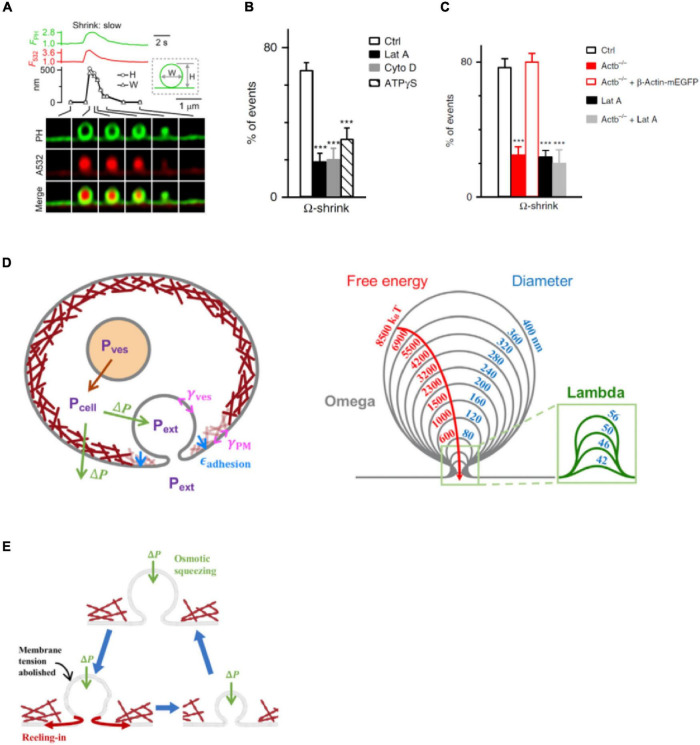
Actin promotes shrink fusion by enhancing plasma membrane tension. **(A)** PH_G_-labeled Ω-profile fluorescence (F_PH_; normalized to baseline), A532 spot fluorescence (F_532_; normalized to baseline), PH_G_-labeled Ω-profile height (H; circles), PH_G_-labeled Ω-profile width (W; triangles), and sampled images at times indicated with lines showing shrink fusion. The experimental setup is the same as that shown in Figure 3E. **(B)** Percentages (mean + SEM) of fusion events undergoing Ω-shrink fusion in Ctrl or in the presence of 3 μM Lat A (***p < 0.001), 4 μM Cyto D (***p < 0.001), or ATPγS (2 mM, replacing 2 mM ATP in the whole-cell pipette; ***p < 0.001) (*t*-test). **(C)** Percentages (mean + SEM) of Ω-shrink fusion induced by whole-cell calcium (1.5 μM) dialysis in Ctrl, Actb^–/–^ cells, Actb^–/–^ cells overexpressed with β-Actin–mEGFP, Ctrl cells treated with Lat A (Lat A), and Actb^–/–^ cells treated with Lat A. ***p < 0.001 (*t*-test). **(D)** Left: schematic of the model (not to scale). Cells maintain an outward (swelling) osmotic pressure ΔP = P_cell_ – P_ext_ (green arrows), with cell pressure P_cell_ exceeding extracellular pressure P_ext_. Intact vesicles maintain swelling pressure (red arrow), with vesicle pressure P_ves_ > P_cell_. Following fusion with plasma membrane (PM), rapid equilibration between vesicle lumen and extracellular medium is assumed, so P_ves_ = P_ext_. The vesicle osmotic pressure then equals ΔP but is now an inward squeezing pressure. The model calculates the vesicle tension, γ_ves_, while the PM tension, γ_PM_, and the adhesion energy ε_adhesion_ to the actin cortex (maroon layer adjacent to PM) are taken from experiment. Right: predicted shrink fusion sequence. Computed vesicle shapes and free energies for squeezing pressure ΔP = 100 Pa and the indicated effective diameters *D* (such that vesicle area equals π*D*^2^). A transition occurs at *D* = 56 nm from Ω to Λ shape (defined as a profile lacking overhang). **(E)** Shrink fusion mechanism predicted by the model. Osmotic squeezing deflates the vesicular Ω-shape profile and abolishes its membrane tension, so the Ω-profile’s membrane is reeled into the PM by PM tension and PM adhesion to the actin cortex. Panels **A,D,E** are adapted from Shin et al. (2020) with permission. Panels **B,C** are adapted from Wen et al. (2016) with permission.

For many decades, two fusion modes were thought to control hormone and transmitter release (Ceccarelli et al., 1973; Heuser and Reese, 1981; Alabi and Tsien, 2013; Wu et al., 2014). One facilitates release *via* fusion pore dilation and flattening, called full-collapse fusion. The other limits release by closing a narrow fusion pore, called kiss-and-run or close-fusion (Ceccarelli et al., 1973; Chiang et al., 2014; Zhao et al., 2016). With super-resolution STED microscopy to visualize fusion modes of dense-core vesicles in neuroendocrine cells, it has been found, surprisingly, that facilitation of release is not mediated by full-collapse, but rather shrink fusion, in which the Ω-profile generated by vesicle fusion shrinks, but maintains a large non-dilating pore until the Ω-profile is undetectable (Figure 4A; Chiang et al., 2014; Wen et al., 2016; Shin et al., 2018, 2021). Inhibition of F-actin polymerization by Lat A, cytochalasin D, or β-actin knockout significantly reduces plasma membrane tension (Figure 3G) and shrink fusion percentage (Figures 4B,C; Wen et al., 2016; Shin et al., 2020). Such an inhibition of shrink fusion can be mimicked by a decrease in plasma membrane tension (*via* increasing the extracellular solution osmolarity) and can be rescued by an increase in plasma membrane tension (*via* decreasing the extracellular solution osmolarity) (Wen et al., 2016). These results suggest that F-actin is essential in mediating shrink fusion (Wen et al., 2016). Furthermore, it has been shown that the F-actin assembly pathway, including N-WASP, formin, and hydrolysis of the energy molecule ATP is involved in mediating shrink fusion (Wen et al., 2016). Inhibition of F-actin leads to accumulation of Ω-shape profiles at the active zone of lamprey synapses, suggesting that F-actin also facilitates merging of fusing vesicles at the plasma membrane, likely also *via* shrink fusion (Wen et al., 2016).

How does F-actin-provided membrane tension mediate shrink fusion? A recent study found that the swelling osmotic pressure maintained by cells, the positive intracellular-to-extracellular osmotic pressure difference, may squeeze the Ω-profile and reduce the Ω-profile membrane tension, generating a tension gradient from the plasma membrane to the Ω-profile that reels Ω-profile membrane into the plasma membrane (Figures 4D,E; Shin et al., 2020). The requirement of the plasma-membrane-to-Ω-profile tension gradient explains why F-actin-dependent plasma membrane tension is needed to mediate shrink fusion (Wen et al., 2016). As the fused vesicle medium equilibrates with the extracellular medium, the squeezing pressure is equal to the swelling osmotic pressure of the cell (Diz-Munoz et al., 2010; Boulant et al., 2011; Stewart et al., 2011; Tsujita et al., 2015; Wen et al., 2016). With squeezing force and membrane-reeling-in force, Ω-profile shrinking is energetically favored over full-collapse fusion (Figures 4D,E), explaining why shrink fusion is selected over full-collapse fusion to merge fusing vesicles at the plasma membrane.

Given that the swelling osmotic pressure and cortical F-actin required for mediating shrink fusion are general properties of cells (Dai and Sheetz, 1999; Diz-Munoz et al., 2010; Boulant et al., 2011; Stewart et al., 2011; Tsujita et al., 2015; Wen et al., 2016), shrink fusion may in fact replace the widely believed-to-be dominant full-collapse fusion in many cell types. It should be noted that as the fusion-generated Ω-profile shrinks at the final stage, the Ω-profile may undergo a transition to a Λ- or dome-shape profile (Shin et al., 2020). This observation has led to the proposal of a shrink-collapse fusion mode, in which Ω-profile shrinking is followed by a transition to a Λ-profile, which in turn is followed by flattening/merging (Shin et al., 2020). This mode unifies the apparent contradiction between shrink fusion and full-collapse fusion.

It has been proposed that small synaptic vesicles, with diameters in the range of ∼20–60 nm, may undergo shrink-collapse fusion, with shrinking as the major component for larger synaptic vesicles and collapse as the primary component for smaller synaptic vesicles. Supporting this proposal, inhibition of F-actin assembly with Lat A, cytochalasin D, or an inhibitor of the formin-dependent F-actin assembly reduces F-actin at nerve terminals and causes accumulation of Ω-profiles at the active zone of lamprey giant synapses (Wen et al., 2016), suggesting that F-actin is also involved in merging small synaptic vesicles, likely *via* facilitating shrink fusion or shrink-collapse fusion.

## Conclusion and Future Research Questions

**FIGURE 5:**
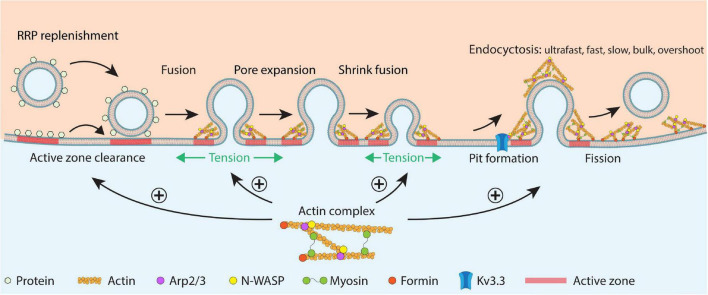
Schematic summary of F-actin’s functions in RRP replenishment, fusion pore expansion, fused vesicle merging *via* shrink fusion, and all distinguishable forms of endocytosis in secretory cells. The schematic drawing shows RRP replenishment involving active zone clearance, vesicle docking and priming, fusion pore opening, fusion pore expansion that releases vesicular contents, shrink fusion that merges fused vesicles at the plasma membrane, and classical endocytosis involving pit formation and fission of the pit. F-actin promotes (1) RRP replenishment likely by facilitating active zone clearance, (2) fusion pore expansion by enhancing the plasma membrane tension, (3) shrink fusion by providing membrane tension to reel of fusing vesicular membrane, and (4) endocytosis likely by generating forces needed for pit formation. Actin nucleation factors, including Kv3.3, N-WASP, Arp2/3, myosin II, and formin are also involved in these processes. This cartoon represents a synthesis of many suggestions derived from the many studies discussed in this review.

Recent studies reveal an essential role of F-actin in mediating all kinetically distinguishable forms of endocytosis, including ultrafast, fast, slow, bulk, and overshoot endocytosis (Figure 5). Ultrastructural examination and real-time imaging suggest that F-actin is involved in pit formation, a critical step of endocytosis that has just been visualized in real time in live cells (Shin et al., 2021, 2022). The essential role of F-actin in mediating endocytosis may facilitate active zone clearance that may in turn promote RRP replenishment (Figure 5). RRP replenishment sustains synaptic transmission and overcomes short-term depression during repetitive firing at synapses. Facilitation of RRP replenishment *via* active zone clearance within seconds after exocytosis is different from the physical barrier function F-actin performs on a much longer time scale during vesicle movement from deep inside the cytosol to the plasma membrane. Such a difference might reconcile the apparent conflict of F-actin’s roles between facilitation of RRP replenishment and being a physical barrier that blocks vesicle movement. Recent studies also showed that F-actin promotes rapid and complete vesicular content release by increasing plasma membrane tension, which facilitates fusion pore expansion (Figure 5). By increasing plasma membrane tension, F-actin reels off fusing vesicle membrane and thus mediates shrink fusion together with the swelling osmotic pressure of the cell that squeezes the fusing Ω-profile (Figure 5). In conclusion, F-actin is essential in (1) mediating all forms of endocytosis and the RRP replenishment that together sustain synaptic transmission, (2) expanding the fusion pore to promote vesicular content release, and (3) mediating shrink fusion or shrink-collapse fusion that merges fusing vesicles with the plasma membrane (Figure 5). Not only actin, but also the actin assembly pathway, including N-WASH-dependent branched F-actin assembly and formin-dependent linear F-actin assembly are involved in mediating these functions of F-actin. A mutation in the Kv3.3 potassium channel, which causes spinocerebellar ataxia 13, inhibits F-actin nucleation, endocytosis, and RRP replenishment and enhances short-term synaptic depression at synapses (Wu et al., 2021), suggesting that impairment of F-actin’s roles in exo- and endocytosis may contribute to neurological disorders.

While recent studies reveal important roles of F-actin in regulating exo- and endocytosis, how F-actin generates forces to mediate endocytosis and pit formation remains not well understood. How actin facilitates active zone clearance and thus RRP replenishment also remains unclear, owing to the difficulty of visualizing active zone clearance in live synapses (Hua et al., 2013). To what extent impairment of F-actin assembly and nucleation plays a role in generating neurological disorders is not well understood. How the cell uses F-actin to shrink fusing Ω-profiles at release sites, but also to generate endocytic pits at endocytic sites, apparently contradictory functions, is also not well understood. It would be of great interest to address these questions in the future.

## Author Contributions

L-GW designed and wrote the manuscript. CYC participated in writing the manuscript. Both authors contributed to manuscript revision, read, and approved the submitted version.

## Conflict of Interest

The authors declare that the research was conducted in the absence of any commercial or financial relationships that could be construed as a potential conflict of interest.

## Publisher’s Note

All claims expressed in this article are solely those of the authors and do not necessarily represent those of their affiliated organizations, or those of the publisher, the editors and the reviewers. Any product that may be evaluated in this article, or claim that may be made by its manufacturer, is not guaranteed or endorsed by the publisher.
